# Endophytic *Bacillus subtilis* antagonize soil-borne fungal pathogens and suppress wilt complex disease in chickpea plants (*Cicer arietinum* L.)

**DOI:** 10.3389/fmicb.2022.994847

**Published:** 2022-11-02

**Authors:** Vellaichamy Mageshwaran, Rishabh Gupta, Shailendra Singh, Pramod K. Sahu, Udai B. Singh, Hillol Chakdar, Samadhan Y. Bagul, Surinder Paul, Harsh V. Singh

**Affiliations:** ^1^Microbial Technology Lab, ICAR-National Bureau of Agriculturally Important Microorganisms, Maunath Bhanjan, Uttar Pradesh, India; ^2^Plant-Microbe Interaction and Rhizosphere Biology Lab, ICAR-National Bureau of Agriculturally Important Microorganisms, Maunath Bhanjan, Uttar Pradesh, India; ^3^ICAR-Directorate of Medicinal and Aromatic Plants Research, Anand, Gujarat, India

**Keywords:** antagonism, bacterial endophytes, *Bacillus subtilis*, chickpea (*Cicer arietinum* L.), fungal pathogens, ISR, seed biopriming, wilt complex

## Abstract

The present study aimed to identify potential endophytic bacteria antagonistic against three soil-borne fungal pathogens, *Rhizoctonia solani, Sclerotium rolfsii,* and *Fusarium oxysporum* f.sp. *ciceri* causing root rot, collar rot, and fungal wilt diseases in chickpea plants, respectively. A total of 255 bacterial endophytes were isolated from the leaves, stems, and roots of seven different crop plants (chickpea, tomato, wheat, berseem, mustard, potato, and green pea). The dual culture-based screening for antifungal properties indicated that three endophytic isolates had strong inhibition (>50%) against all three pathogens tested. Based on morphological, biochemical, and molecular characterization, the selected isolates (TRO4, CLO5, and PLO3) were identified as different strains of *Bacillus subtilis*. The bacterial endophytes (TRO4 and CLO5) were positive for plant growth promoting (PGP) traits *viz.*, ammonia, siderophore, and indole-3-acetic acid (IAA) production. The bio-efficacy of the endophytes (TRO4, CLO5, and PLO3) was tested by an *in planta* trial in chickpea pre-challenged with *R. solani, S. rolfsii,* and *F. oxysporum* f.sp. *ciceri*. The *B. subtilis* strains TRO4 and CLO5 were found to be effective in reducing percent disease incidence (*p* ≤ 0.05) and enhancing plant growth parameters. The different root parameters *viz.* root length (mm), surface area (cm^2^), root diameter (mm), and root volume (cm^3^) were significantly (p ≤ 0.05) increased in TRO4 and CLO5 inoculated chickpea plants. Confocal Scanning Laser Microscopy showed heavy colonization of bacteria in the roots of endophyte-inoculated chickpea plants. The inoculation of endophytic *Bacillus subtilis* strains TRO4 and CLO5 in chickpea plants through seed biopriming reduced the accumulation of superoxide, enhanced the plant defense enzymes, and induced the expression of Pathogenesis-Related (PR) genes. Semi-quantitative analysis of defense-related genes showed differential activation of PR genes (*60srp* and *IFR*) by endophyte inoculation. The results of the present study reveal the antagonistic potential of *B. subtilis* strains TRO4 and CLO5 against three major soil-borne fungal pathogens and their ability to suppress wilt complex disease in chickpea plants. This is the first report on the simultaneous suppression of three major soil-borne fungal pathogens causing wilt complex in chickpea plants by endophytic *B. subtilis* strains.

## Introduction

Chickpea (*Cicer arietinum* L.) is the major food legume grown in south Asia. Globally, it is the third largest legume crop grown after common bean and field pea (Mula et al., 2011). Chickpea has been grown in the area of 14.56 million ha with a productivity of 970 kg ha^−1^. India is the largest producer with 65% of the global production. The other major chickpea-producing countries are Pakistan, Turkey, Iran, Myanmar, Australia, Ethiopia, Canada, Mexico, and Iraq. It is a major pulse crop in India with a share of 46% followed by Pigeonpea, Urd bean, Mung bean, Lentil, Pea, and others (Merga and Haji, 2019). In general, chickpea production is largely affected by five important soil-borne phytopathogens that cause wilt and root rot complex in chickpea plants. Among them, fusarium wilt, dry root rot, wet root rot, black root rot, and collar rot are most important and caused by *Fusarium oxysporum* f.sp. *ciceri*, *Rhizoctonia bataticola*, *R. solani*, *F. solani*, and *Sclerotium rolfsii*, respectively (Beniwal et al., 1992). The soil-borne plant pathogenic fungi *viz.*, *F. oxysporum*, *S. rolfsii*, and *R. solani* have a wide host range, occur in combination, and cause severe disease complex symptoms which in turn lead to great worldwide economic losses every year (Rudresh et al., 2005; Senthilkumar et al., 2009). *R. solani* causes root rot in chickpea/sugar beet, bare patch in cereals, black scurf in potatoes, and sheath blight in rice. They infect the lower root and stem of the plant (Anderson, 1982). However, *Sclerotium rolfsii* causes collar rot disease in chickpea, watermelon, pepper, tomato, sweet potato, onion, and groundnut. They infect the host in the early stages of crop growth. White threads of mycelial growth in a fan shape pattern on the stem and plant leaves are a sign of this disease (Aycock, 1966; Sahu et al., 2019). *Fusarium oxysporum* f.sp. *ciceri* is a soil-borne pathogen and causes wilt disease in chickpea plants. It is a devastating disease and can cause a yield loss of 10–100% in chickpea depending on the aggressiveness of fungal inoculum, virulence, and environmental conditions. The pathogen is a facultative saprophyte and can survive in soil or crop residues as chlamydospores for up to 6 years (Agrios, 2005). The unique symptoms are the drooping of the petioles, rachis, and leaves, and internal discoloration (browning) of xylem vessels (Jendoubi et al., 2017).

Management of the wilt complex is largely dependent on the use of toxic chemical fungicides. These chemicals are toxic to non-target flora and fauna in the soil ecosystem (Singh et al., 2016). Further, the residual impact of these toxic chemicals on the health of animals, humans, and the environment. The breeding of resistant cultivars is one of the safer alternatives and to date, some cultivars are available with moderate resistance to either of these pathogens. However, the availability of suitable donor parents with a high degree of resistant genes/quantitative trait loci and the transfer of these traits to agronomically important cultivars is a great challenge for plant breeders (Singh et al., 2016). Looking at the importance of the crops and the problem therein, biological control of these notorious pathogens is a viable and sustainable management option as chemical fungicides harm the environment and human health (Sarma et al., 2015; Singh et al., 2020a). In the recent past, several researchers have reported that plant growth promoting rhizobacteria (PGPR) mediates modulation of systemic resistance against wilt and root rot pathogens in many crops including chickpea (Rudresh et al., 2005; Zaim et al., 2016; Bekkar et al., 2018; Kumari and Khanna, 2019). Besides PGPRs, bacterial endophytes are a group of bacteria that have a special ability to colonize and reside inside the plants and protect the host plant from biotic and abiotic stresses (Ziedan, 2006; Sahu et al., 2020, 2021; Singh et al., 2021). Among the plant parts, the roots are considered the major entry point of microorganisms and have the highest frequency of colonization of endophytic bacteria. Bacterial endophytes colonize the same niche as plant pathogens and suppress the pathogen through various mechanisms such as the production of hydrogen cyanide (HCN), siderophore, hydrolytic enzymes, antibiotics, and induction of systemic resistance (Sahu et al., 2020; Singh et al., 2020a,b). Further, they support plant growth through the production of phytohormones, solubilizing nutrients, and nitrogen fixation (Ryan et al., 2007; Senthilkumar et al., 2009). Thus, bacterial endophytes could be used as an efficient bio-control agent against potential soil-borne pathogens as they provide localized protection to the host plant. Beneficial bacteria living inside the plant interact with the host and fight against different phytopathogens (Sahu et al., 2019, 2020). These microorganisms either produce broad-spectrum biotic stimuli or directly interact with the pathogens during the infection and/or invasion process and thereby enhance the physiological state of defense, known in general as induced systemic resistance. The induction of systemic resistance is categorized as (i) induced systemic resistance (ISR) and (ii) systemic acquired resistance (SAR). ISR occurs when plants’ intrinsic defense mechanisms are triggered in response to biotic threats (Pineda et al., 2013; Sahu et al., 2020; Singh et al., 2020a). In general, during host-pathogen interaction, microbial inoculants activate the various defense enzymes (PPO, POX, GLU, CHI, and PAL) and several other pathways/cascades responsible for systemic resistance in plants such as phenylpropanoid, MAPKs, and jasmonate, (Harman et al., 2004; Harman, 2011; Sahu et al., 2020; Singh et al., 2020a). Reduction in the disease severity and enhanced up-regulation and bioaccumulation of defensive enzymes triggered by the combined inoculation of *Bacillus atrophaeus*, *B. subtilis*, and *Burkholderia cepacia* exhibited a direct bio-control and ISR in the suppression of vascular disease in tomato crops (Shanmugam and Kanoujia, 2011). Although the isolation of bacterial endophytes and their antagonist property against fungal pathogens have been reported so far (Souza et al., 2014; Gond et al., 2015; Zhao et al., 2015), there is no report on the bio-control of pathogens involved in wilt complex disease in chickpea using bacterial endophytes. Looking at the importance of root diseases especially, wilt complex in chickpea and endophytes with antimicrobial properties, it is the need of the hour to explore the potential of endophytes for the management of wilt complex in chickpea. It is hypothesized that bacterial endophytes provide localized and systemic protection against invasion of soilborne fungal pathogens, Therefore, the present investigation was aimed to identify bacterial endophytes showing strong antagonism against multiple plant pathogenic fungi, *R. solani, S. rolfsii,* and *F. oxysporum* f.sp. *ciceri* and bio-control of wilt complex disease caused by these pathogens in chickpea using selected potential endophytes.

## Materials and methods

### Collection of plant samples

The whole plant samples (chickpea, tomato, wheat, berseem, mustard, potato, and green pea) were collected from different agricultural farms in a village, Onhaich, which is situated at nearby ICAR-National Bureau of Agriculturally Important Microorganisms (NBAIM), Mau, Uttar Pradesh, India (25°53′30”N; 83°28′46″ E). The sampling was done in the month of January 2020. The crops were of local commercial cultivars and were approximately 20–30 days old during sample collection. The plant parts *viz.*, roots, stems, and leaves were separated and washed carefully in running tap water. Thereafter, the clean samples were used immediately for the isolation of bacterial endophytes.

### Microorganisms

The test fungal pathogens, *R. solani*, *S. rolfsii* were obtained from the Plant-Microbe Interaction Laboratory and Rhizosphere Biology Lab, ICAR-NBAIM, Kushmaur, whereas *F. oxysporum* f.sp. *ciceri* (NAIMCC-F-02214) was obtained from the National Agriculturally Important Microbial Culture Collection (NAIMCC), ICAR-NBAIM, Kushmaur. The fungal cultures were grown on the Petri plates containing potato dextrose agar (PDA, HiMedia Pvt. Ltd., Mumbai, India) at 28 ± 2°C till complete growth. The full-grown fungal cultures were preserved at 4°C till further use.

### Isolation of bacterial endophytes

The roots, stems, and leaves (1 g each) were cleaned in running water to remove adhering particles. The endophytic bacterial strains were isolated from different plant samples using the protocols described by Sahu et al. (2020) with slight modifications. Briefly, the plant parts were surface sterilized with 70% ethanol for 30 s in the case of the leaves, 1 min for the stems, and 2 min for the roots, and all were subsequently washed with sterile water thrice. Thereafter, the plant parts were surface sterilized with 2% sodium hypochlorite for 30 s in the case of the leaves, 1 min for the stems, and 1.5 min for the roots. To ascertain the successful sterilization, the representative surface sterilized plant parts were placed at the center of nutrient agar (NA, HiMedia Pvt. Ltd., Mumbai, India) plates and incubated at 30°C for 48 h to observe the presence or absence of microbial growth on the plant surface. The surface sterilized samples were cut into small pieces and crushed along with 10 ml of 0.8% saline water in a sterile pestle and mortar. The dilutions (10^−1^, 10^−2^, and 10^−3^) of the samples were made using 0.8% saline water. Each dilution of the sample (0.1 ml) was spread over NA, potato dextrose agar (PDA), R-2A, and Luria Bertani agar (LBA, HiMedia Pvt. Ltd., Mumbai, India) plates to obtain the endophytic bacteria diversity. The composition (g/L) of R-2A agar medium used as follows: Enzymatic digest of casein – 0.25; Enzymatic digest of animal tissue – 0.25; Acid hydrolysate of casein – 0.5; Yeast extract – 0.5; Glucose – 0.5; Starch soluble – 0.5; Dipotassium phosphate – 0.3; Magnesium sulfate heptahydrate – 0.05; Sodium pyruvate – 0.3; Agar-15. The plates were incubated at 30°C for 24 h. After incubation, the bacterial colonies with distinct colony morphology were subcultured on NA plates to obtain pure colonies. The pure cultures were maintained in the refrigerator at 4°C until further use.

### Selection of potential antagonist(s)

During isolation, 255 bacterial isolates were obtained from different plant samples used in the present study. These isolates were preliminarily screened for antagonistic activity against fungal pathogens, *R. solani*, *S. rolfsii*, and *F. oxysporum* using a dual culture plate assay (Singh et al., 2016). Briefly, the fungal disc (5 mm diameter) was made by unplugging the fully grown fungal pathogens in PDA plates using the flame sterilized cork borer. The fungal disc was kept at the center of a fresh PDA plate and the bacterial endophytes to be tested were streaked on side of the Petri plate. The inoculated plates were kept for incubation at 28 ± 2°C for 7 days. After 7 days of incubation, the plates were observed for any inhibitory zones. The bacterial endophytes showing strong inhibition (>5 mm radius of zone of inhibition) against all the test fungal pathogens were selected for further characterization.

### Morphological and biochemical characterization of the selected bacterial endophytes

The morphological and biochemical characterization of the selected bacterial endophytes (TRO4, CLO5, and PLO3) was performed using standard protocols. To designate the strain number, the following rule was adopted: the first letter stands for the crop type (T-Tomato; C-Chickpea; P-Potato), the second letter stands for plant part (R-Root; L-Leaf) and the third letter stands for the place the crop was grown (O – Onhaich). The morphological parameters such as colony characteristics, Gram staining, and endospore staining and the biochemical characteristics such as catalase, oxidase, nitrate reductase, starch hydrolysis, Methyl Red Voges Proskaeur (MR-VP), indole production, citrate utilization, urease production, and hydrogen sulfide production were assessed according to Dubey and Maheswari (2008).

### Evaluation of growth characteristics

The growth characteristics of the selected endophytes (TRO4, CLO5, and PLO3), such as specific growth rate and generation time, were determined according to Mageshwaran et al. (2014). Each bacterial culture was inoculated in a nutrient broth (1X) in 5 wells of 96-well microtitre plates and incubated for 72 h at 30°C in a growth kinetics chamber (Bioscreen C, Clover Scientific Pvt. Ltd., New Delhi) and the absorbance at 600 nm was recorded at 1-h intervals.

### Characterization of selected strains for PGP traits

The PGP traits such as ammonia production, mineral solubilization (phosphate, potassium, and zinc), siderophore production, and hydrogen cyanide (HCN) were assessed in the selected strains. The selected bacterial isolates grown in a nutrient broth for 24 h were characterized for PGP traits qualitatively using the methods as described by Sharma et al. (2016) with slight modification (Singh et al., 2016).

### Quantitative determination of antifungal property by dual culture assay

The selected bacterial endophytic isolates (TRO4, CLO5, and PLO3) were evaluated quantitatively for antifungal properties. The fungal disc was kept at the center of a fresh PDA plate and the selected bacterial endophyte was streaked (co-inoculate) on both sides of the Petri plates equidistant from the disc and incubated at 28 ± 2°C for 48 h. The inhibition percentage was calculated using the formula: inhibition of mycelial growth percentage = (A-B)/A × 100, where A is the diameter of mycelial growth of the fungal pathogen in the control plate, B is the diameter of mycelial growth of the fungal pathogen in the dual culture plate.

### Molecular characterization of selected bacterial endophytes based on 16s rRNA gene sequencing

Bacterial endophytes were grown in NB at 28 ± 2°C for 24 h, centrifuged, and the pellets were separated. The pellets were used for DNA extraction. Genomic DNA of bacterial endophytes was extracted using Nucleopore gDNA Fungal Bacterial mini kit (Genetix Biotech Asia Pvt. Ltd., India, NB-7006 D) following the manufacturer protocols. To identify the selected bacterial endophytic isolates at the species level, PCR amplification of the 16s rRNA gene was done using the GoTaq Green master mix (M/s Promega, United States). The primer pair used in the PCR was: forward primer 27 F(AGA GTT TGA TCC TGG CTC AG) and reverse primer 1,492 R (TAC GGT TAC CTT GTT ACG ACT; GeNei, India). The PCR amplification for 16s rRNA (35 cycles) was done using a thermocycler (PeqSTAR, VWR, United States) and the PCR conditions were as follows: heat lid temperature 110°C, initial denaturation temperature 94°C for 4 min, denaturation temperature 94°C for 30 s, annealing temperature 52°C for 45 s, extension temperature 72°C for 90 s, and final extension temperature 72°C for 10 min. The PCR product was run in 1.2% agarose gel electrophoresis along with a 1 kb DNA ladder (Thermo Fisher Scientific, United States). The intact DNA band visible near 1,500 bp was cut with the help of a sterile scalpel blade and transferred into a 2 ml centrifuge tube. The 16s rRNA amplicons were purified with the help of a DNA purification kit (Nucleopore, Genetix Biotech Asia (P) Ltd., New Delhi). The purified 16s rRNA was sequenced using a 16 capillary ABI sequencer (ABI prism 3130XL) and sequence quality was checked using FinchTV software. The contigs were made using Bioedit software. The 16s rDNA sequences were BLAST in the NCBI database to obtain the identity of the cultures by finding the closest related species.

### *In vivo* evaluation of selected endophytes for growth and antagonism against wilt complex disease in chickpea

The shortlisted endophytic *Bacillus subtilis* strains (TRO4, CLO5, and PLO3) were further evaluated for growth and antagonism against wilt complex disease in chickpea plants. The present investigation was based on previous baseline studies which confirmed the suppression of pathogens by the selected endophytes. Pot culture experiments were conducted from October to December 2020 (winter season) at ICAR-NBAIM, Mau.

#### Preparation of inoculum

The inoculum of fungal pathogens was developed by inoculating the mother culture in a flask containing the autoclaved (two times) sand and maize grains at 8:1 (w/w) according to Rudresh et al. (2005) with a slight modification in that maize grains were used in place of sorghum. The inoculated flasks were incubated for 2 weeks at 28 ± 2°C and used for inoculating soil. The inoculum of endophytic bacteria was prepared by inoculating the culture (from agar plates) to the flask containing 100 ml of sterilized nutrient broth (1X) and incubated for 24 h under shaking conditions (125 rpm) at 28 ± 2°C. The colony-forming unit of the inoculum was calculated as 1 × 10^8^ CFU ml^−1^.

#### Experimental set-up

The experimental soil was collected from the agricultural farm, ICAR-NBAIM, Mau. The physicochemical properties of the soil used in the experiment were pH (8.0), electrical conductivity (0.81 dS/m), and organic carbon (0.39%). The experimental soil was moistened and autoclaved twice at 12 h intervals. Clean plastic pots (9 × 15 cm) were three-quarters filled with sterile soil. Each pot contained 2 kg of sterile soil. The fungal inoculum (10 g) with a colony-forming unit of 1.25 × 10^4^ CFU g^−1^ was added to the pots and mixed properly. The inoculated pots were incubated for 10 days to develop and establish the fungal inoculum in the pots containing experimental soil (Rudresh et al., 2005). Seed priming with endophytes was done by suspending the surface sterilized chickpea seeds (susceptible cv. JG 62) in endophytes inoculum for 30 min according to Singh et al. (2021). Five seeds were sown in each pot.

Three sets of experiments were conducted to evaluate the biocontrol efficacy of bacterial endophytes against three soil-borne pathogens. The first set of experiments consisted of treatments to evaluate the biocontrol against *R. solani,* while the second set of experiments consists of treatments to evaluate the biocontrol against *S. rolfsii* and the third set of experiments consists of treatments to evaluate the biocontrol against *F. oxysporum* f.sp. *ciceri* (Table 1). A negative control [Uninoculated (UC)] was maintained in which neither pathogen nor endophyte was inoculated. Plants were irrigated with Jenson’s liquid nutrient medium (Senthilkumar et al., 2009) once a week for 45 days. Five replications were maintained for each treatment. The experiment was conducted in a completely randomized design (CRD).

**Table 1:** Treatments detail.

S. No.	Treatments		
	Set-1: *R. solani*	Set-2: *S. rolfsii*	Set-3: *F. oxysporum* f.sp*. ciceri*
1.	A1: positive control (inoculated with *R. solani* alone)	B1: positive control (inoculated with *S. rolfsii* alone)	C1: positive control (inoculated with. *F. oxysporum* f.sp*. ciceri* alone)
2.	A2: inoculated with *B. subtilis* TRO4 + *R*. *solani*	B2: inoculated with *B. subtilis* TRO4 + *S. rolfsii*	C2: inoculated with *B. subtilis* TRO4 + *F. oxysporum* f.sp*. ciceri*
3.	A3: inoculated with *B. subtilis* CLO5 + *R. solani*	B3: inoculated with *B. subtilis* CLO5 + *S. rolfsii*	C3: inoculated with *B. subtilis* CLO5 + *F. oxysporum* f.sp.*ciceri*
4.	A4: inoculated with *B. subtilis* PLO3 + *R. solani*	B4: inoculated with *B. subtilis* PLO3 + *S. rolfsii*	C4: inoculated with *B. subtilis* PLO3 + *F. oxysporum* f.sp*. ciceri*
5.	A5: Carbendazim at 2 g/kg of seeds + *R. solani* (chemical control).	B5: Carbendazim at 2 g/kg of seeds + *S. rolfsii* (chemical control)	C5: Carbendazim at 2 g/kg of seeds + *F. oxysporum* f.sp*. ciceri* (chemical control)

Further, the best performing endophytic *Bacillus subtilis* strains, TRO4 and CLO5 were evaluated for the induction of ISR in chickpea plants. There were three sets of experiments to evaluate the induction of ISR by bacterial endophytes in chickpea plants pre-challenged with soil-borne fungal pathogens. Each set of experiments consists of five treatments. For all the experiments, a negative control (Uninoculated (UC)) in which neither pathogen nor endophyte was inoculated. The resistant cultivar (RC; var. Avrodhi) was taken as a standard check. The first set of experiments consists of treatments pre-challenged with *R. solani*-A1 positive control (inoculated with *R. solani* alone), A2 inoculated with *B. subtilis* TRO4 + *R*. *solani* and A3 inoculated with *B. subtilis* CLO5 + *R. solani*. The second set of experiments consists of treatments to evaluate the biocontrol of *S. rolfsii-B1* positive control (inoculated with *S. rolfsii* alone), B2 inoculated with *B. subtilis* TRO4 + *S. rolfsii*, and B3 inoculated with *B. subtilis* CLO5 + *S. rolfsii*. The third set of experiments consists of treatments to evaluate the biocontrol of *F. oxysporum* f.sp. *ciceri* – C1 positive control (inoculated with. *F. oxysporum* f.sp*. ciceri* alone), C2 inoculated with *B. subtilis* TRO4 + *F. oxysporum* f.sp*. ciceri*, and C3 inoculated with *B. subtilis* CLO5 + *F. oxysporum* f.sp. *ciceri*. Five replications were maintained for each treatment. The experiment was conducted in a completely randomized design (CRD).

#### Assessment of disease incidence and plant growth

Germination (%) was recorded 7 days after sowing. The plants were observed for the appearance of disease symptoms at 15, 30, and 45 days after sowing (DAS). A numerical disease rating was assigned as follows: 0-healthy seedlings; 1-brown lesions on collar/root region; 3-stunted growth; 4-dead plants with completely dried leaves. Mean Disease Rate (MDR) and Percentage Disease Incidence (PDI) of germinated plants were calculated according to the formula described by Senthilkumar et al. (2009) as given here.

$$\begin{array}{l} MDR=\left[\left(a*0\right)+\left(b*1\right)+\left(c*2\right)+\left(d*3\right)+\left(e*4\right)\right]\\ \;/\left(a+b+c+d+e\right) \end{array}$$

where *a*, *b*, *c*, *d*, and *e* are the number of plants with a disease rating of 0, 1, 2, 3, and 4, respectively.

$$PDI=\frac{MDR*100}{4\;\left(\mathrm{maximum grade}\right)}$$

At 45 DAS, the plants were uprooted and the roots were washed using slow-running tap water and the observations such as plant height (cm), the number of branches per plant, and dry weight of plant biomass (after drying in an oven at 60°C for 6 h) were recorded using standard protocols.

### Analysis of root morphology

Forty-five days after sowing, chickpea plants from three replicates of all the treatments were uprooted and washed, and cleaned roots were used to study the root architecture. The root parameters such as root length (mm), surface area (cm^2^), the average diameter (mm), and root volume (cm^3^) were determined using a Hewlett Packard scanner and analyzed using the WinRHIZO V. 2002\u00B0C software (Regent Instruments Inc. Ltd. Quebec, Canada) according to Singh et al. (2017).

### Confocal scanning laser microscopic analysis

A separate experimental trial was conducted under gnotobiotic conditions to evaluate the colonization potential of bacterial endophytes in chickpea plants. The colonization pattern of *B. subtilis* strains TRO4 and CLO5 in 45-day-old chickpea roots was examined under the Confocal Scanning Laser Microscope (CSLM; Nikon Eclipse 90i). Freshly collected roots were stained with LIVE/DEAD™ BacLight bacterial viability stain (Invitrogen, United States) to localize the colonization of inoculated bacteria as described by Sahu et al. (2019). The colonization was compared with control plant roots with similar optical adjustments. The images were processed using NIS Element 3.2.3 software (Nikon, Japan).

### *In vivo* evaluation of bacterial endophytes on induction of ISR in chickpea

#### Effect of endophytes inoculation on the accumulation of superoxide radicals

The accumulation of superoxide radicals (O^2−^) in the leaf was visualized under a stereomicroscope after staining with nitroblue tetrazolium (NBT) as described by Sahu et al. (2019). The leaves collected from chickpea plants pre-challenged with *R. solani* after 45 DAS were stained with NBT and the formation of blue color formazan was taken as an indication of superoxide accumulation.

#### Effect of endophytes inoculation on plant defense-related enzymes

To evaluate the effect of endophytes inoculation on ISR in chickpea plants, defense-related enzymes *viz.*, phenylalanine ammonia-lyase (PAL) and peroxidase (PO) were assayed (Sadasivam and Manickam, 1996). For estimation of PAL activity, a leaf tissue sample (0.5 g) was extracted in 4 ml of 0.2 M borate buffer (pH 8.7) with 1.4 mM β-mercaptoethanol. Enzyme extract (200 μl) was added with 500 μl borate buffer, 1 ml of 0.1 M L-phenylalanine, and 1.3 ml of distilled water. The mixture was incubated at 32°C for 30 min and the reaction was terminated by adding 500 μl of Trichloroacetic acid. The activity was measured at 290 nm and expressed in μmole of *trans*-cinnamic acid g^−1^ fresh weight. For the estimation of PO activity, 0.5 g of leaf tissue was extracted in 4 ml of 50 mM phosphate buffer. Enzyme extract (200 μl) was added with 3 ml of 50 mM phosphate buffer, 0.5 ml of 20 mM guaiacol, and 300 μl of 12.3 mM H_2_O_2_. The absorbance was measured at 436 nm.

#### Effect of endophytes inoculation on the expression of PR genes

The expression of PR genes (*60srp* and *IFR*) was studied using the semi-quantitative reverse transcriptase PCR (semi-q RT-PCR) along with the housekeeping gene CHS, CAC and GADPH. The pathogen was inoculated and plants were raised as described in section 2.9. Total RNA isolation, cDNA synthesis, and PCR amplification of housekeeping and PR genes were performed according to Sahu et al. (2019). The primer sequences for PCR amplification of defense-related chickpea genes are given in Supplementary Table 3.

### Statistical analysis

The data collected in this study were analyzed in a completely randomized design (CRD) using a one-way analysis of variance (ANOVA; WASP.1; ICAR research complex, Goa). For all analyses, the differences were considered to be significant at *p* ≤ 0.05.

## Results

### Assessment of bacterial endophytes for antagonistic activity and PGP traits under *in vitro* conditions

In the present study, an attempt was made to isolate bacterial endophytes from surface-sterilized plant parts of seven different crop plants. The successful sterilization of plant parts was ascertained by the absence of any microbial growth in the surface-sterilized plant part kept on NA medium (data not shown). In total, 255 bacterial endophytes were isolated from different plant parts (stems, leaves, and roots) of the collected plant samples. Among the crop plants, the greatest number of isolates was obtained from wheat (54), followed by chickpea (49), berseem (41), potato (40), tomato (30), mustard (27), and green pea (14). Among the plant parts, the greater number of isolates was obtained from the roots (91), followed by the stems (84), and leaves (80; Table 2). All the isolated bacterial endophytes were subject to screening for antifungal properties against all three pathogens on a dual plate culture assay. The number of isolates showing inhibition against *R. solani*, *S. rolfsii*, and *F. oxysporum* f.sp. *ciceri*, individually, and all three pathogens simultaneously, were 55, 45, 16, and 16, respectively. Out of which, the number of isolates showing inhibition of more than a 5 mm radius was 16, 15, 6, and 3, each pathogen and all three, respectively (Table 2). The three bacterial isolates showing inhibition (>5 mm radius) against all three test fungal pathogens were considered for further experiments.

**Table 2:** Screening of bacterial endophytes against soil-borne fungal pathogens.

Crop	Plant part	No. of total isolates	Number of isolates antagonistic against			
			*R. solani*	*S. rolfsii*	*F. oxysporum* f.sp. *ciceri*	All the three pathogens
Chickpea	Leaf	15	06 (02)	05 (02)	03 (01)	03 (01)
	Stem	19	09 (02)	–	–	–
	Root	15	01	–	–	–
Tomato	Leaf	08	03 (03)	01	01 (01)	01
	Stem	16	–	02	–	–
	Root	06	03 (02)	04 (02)	03 (02)	03 (01)
Wheat	Leaf	09	–	03	–	–
	Stem	13	04	02	02	02
	Root	32	03	03 (01)	02	02
Berseem	Leaf	15	06	02	01	01
	Stem	10	05 (03)	04 (04)	–	–
	Root	16	03	02	–	–
Mustard	Leaf	12	1	–	–	–
	Stem	08	–	01	–	–
	Root	07	02 (01)	03 (02)	02	02
Potato	Leaf	14	02 (02)	03(02)	02(02)	02(01)
	Stem	17	01	02	–	–
	Root	09	06 (01)	02	–	–
Green pea	Leaf	07	–	06 (02)	–	–
	Stem	01	–	–	–	–
	Root	06	–	–	–	–
Total		255	55 (16)	45 (15)	16 (06)	16 (03)

The value in the brackets shows the number of isolates showing strong inhibition (>5 mm radius of zone of inhibition) against the pathogen.

Thus, out of 255 bacterial isolates, 3 isolates (TRO4, CLO5, and PLO3) showed strong antagonism against all three fungal pathogens, *R. solani*, *S. rolfsii*, and *F. oxysporum* f.sp. *ciceri*. The biochemical characterization showed that all three selected bacterial endophytes were positive for Gram staining, endospore staining, catalase, oxidase, nitrate reductase, starch hydrolysis, Voges-Proskauer, and citrate utilization while they were negative for methyl red, indole, urease, and H_2_S production test. Morphologically, the colonies were a cream color, rough, circular, entire surface, and opaque (results not shown). The results of morphological and biochemical characterization showed the selected bacterial endophytes belong to *Bacillus* sp. The specific growth rate (μ) of the endophytes was 0.38 (h^−1^) for TRO4 and CLO5 and 0.43 (h^−1^) for PLO3. The molecular characterization of 16s rRNA sequences and identification based on the closest related species obtained from the NCBI database revealed that the selected bacterial endophytes belong to different strains of *B. subtilis* (Table 3). The 16s rRNA sequences were submitted to the NCBI database and the GenBank accession numbers were obtained for bacterial endophytes (Table 3). The bacterial endophytes (TRO4, CLO5, and PLO3) were submitted to the National Agriculturally Important Microbial Culture Collection (NAIMCC), Mau, India, and the accession numbers were obtained (Table 3).

**Table 3:** Molecular characterization of the selected bacterial endophytes based on 16s rRNA gene sequence analysis.

Isolate	GenBank accession number	Closest related species	% of similarity	Query size (base pair)	NAIMCC accession no.
TRO4	MW888880	*Bacillus subtilis* strain SEGB1 (MN565269.1)	100	1,396	B-02794
CLO5	MW888882	*Bacillus subtilis* strain soil G2B (MT641205.1)	100	1,405	B-02790
PLO3	MW888883	*Bacillus subtilis* sub sp. stercosis (MN704443.1)	100	1,401	B-02792

**Figure 1:**
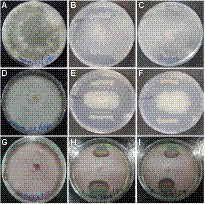
Antagonistic property of endophytic *Bacillus subtilis* strains TRO4 and CLO5 against soil-borne fungal pathogens. **(A)** *R. solani* control, **(B)** *R. solani* vs. TRO4, **(C)** *R. solani* vs. CLO5, **(D)** *S. rolfsii* control, **(E)** *S. rolfsii* vs. TRO4, **(F)** *S. rolfsii* vs. CLO5, **(G)** *F. oxysporum* control, **(H)** *F. oxysporum* vs. TRO4, **(I)** *F. oxysporum* vs. CLO5.

The endophytic *B. subtilis* strains (TRO4, CLO5, and PLO3) were assessed for their antagonistic property by evaluating the inhibition of fungal growth percentage using a dual culture assay and various plant growth promoting characteristics. All the selected endophytes showed more than 50% inhibition of the tested pathogens in a dual plate assay. The bacterial endophytes (CLO5 and PLO3) showed a higher inhibition percentage (64.3%) against *R. solani*, whereas TRO4 had a higher inhibition percentage (83.8 and 70.9%) against *S. rolfsii* and *F. oxysporum* f.sp. *ciceri,* respectively (Table 4). The qualitative determination of plant growth-promoting traits showed that all the selected endophytes were positive for ammonia and siderophore production. The bacterial endophytes, TRO4 and CLO5 were positive for IAA production. All the selected endophytes were negative for mineral solubilization (phosphate, potassium, and zinc), chitinase activity, and HCN production. The antifungal activity of TRO4 and CLO5 against all three fungal pathogens tested is depicted in Figure 1.

**Table 4:** Antifungal properties and PGP of selected bacterial endophytes.

Isolate	% of inhibition			Plant Growth Promoting traits							
	*Rhizoctonia solani*	*Sclerotium rolfsii*	*Fusarium oxysporum*	I	II	III	IV	V	VI	VII	VIII
TRO4	58.9	83.8	70.9	+	−	−	−	+	−	−	+
CLO5	64.3	73.3	69.2	+	−	−	−	+	−	−	+
PLO3	64.3	62.9	66.5	+	−	−	−	+	−	−	−

Ammonia production (I), Phosphate solubilization (II), Potassium solubilization (III), Zinc solubilization (IV), Siderophore Production (V), HCN production (VI), Chitinase production (VII), IAA production (XIII). The (+) and (−) signs indicate positive and negative for PGP traits, respectively.

### Bio-efficacy of endophytes on growth and antagonism against wilt complex

**Figure 2:**
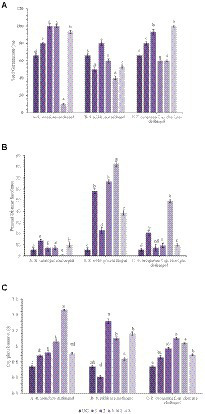
Effect of endophytes inoculation on plant growth and disease incidence in chickpea [**A**, Seed Germination (%); **B**, Percent Disease Incidence; **C**, Dry plant biomass (g)]. Treatment details: UC-Uninoculated (negative control which is not inoculated with a pathogen or bacterial strain). A1, B1, C1 – Positive control of *R. solani*, *S. rolfsii,* and *F. oxysporum* f. sp. *ciceri*, respectively in which only pathogen inoculated. A2, B2, C2-Respective pathogen + *B. subtilis* strain TRO4 inoculated. A3, B3, C3-Respective pathogen + B*. subtilis* strain CLO5 inoculated. A4, B4, C4-Respective pathogen + *B. subtilis* strain PLO3 inoculated. A5, B5, C5-Chemical control (Respective pathogen + Carbendazim at 2 g per kg of seed). The PDI was calculated based on the number of plants germinated. Data are mean (n = 5) and vertical bars represent standard deviation.

**Figure 3:**
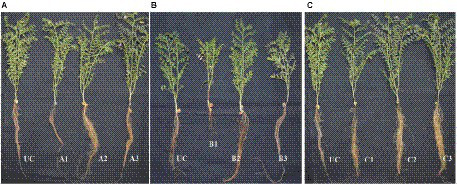
Effect of bacterial endophytes on suppression of root wilt complex disease in chickpea plants (**A**, *R. solani* pre-challenged; **B**, *S. rolfsii* pre-challenged; **C**, *F. oxysporum* f. sp. *ciceri* pre-challenged). Treatment details: UC-Uninoculated (negative control which is not inoculated with a pathogen or bacterial strain). A1, B1, C1 – Positive control of *R. solani*, *S. rolfsii,* and *F. oxysporum* f. sp. *ciceri*, respectively in which only pathogen inoculated. A2, B2, C2 – Respective pathogen + *B. subtilis* strain TRO4 inoculated. A3, B3, C3 – Respective pathogen + *B. subtilis* strain CLO5 inoculated. The age of the plant under observation was 45 days after sowing.

A pot culture experiment was conducted in the winter season from November–December, 2020 to evaluate the effect of seed priming with bacterial endophytes on plant growth and the suppression of wilt complex disease in chickpea plants. Three sets of experiments (one set for each pathogen) were conducted to evaluate the ability of the chickpea plants to suppress root rot caused by *R. solani*, collar rot caused by *S. rolfsii,* and wilt caused by *F. oxysporum* f.sp. *ciceri* using bacterial endophytic strains (TRO4, CLO5, and PLO3). The plant growth characteristics such as plant height, dry plant biomass, and the number of branches per plant were recorded at 45 DAS. Germination percentage was recorded at 10 DAS. The disease incidence was recorded at 15, 30, and 45 DAS. The results on the effect of bacterial endophytes on germination percentage, percent disease incidence, and dry plant biomass (g plant^−1^) at 45 DAS are given in Figure 2. The MDR and PDI recorded at 15, 30, and 45 days are given in Supplementary Table 1. The effect of bacterial endophytes on plant height and the number of branches per plant is given in Supplementary Table 2. The suppression of soil-borne fungal pathogens by bacterial endophytes (TRO4 and CLO5) in chickpea plants (45 days old) is pictorially depicted in Figure 3.

#### *Rhizoctonia solani* pre-challenged soil

Germination percentage was higher in the bacterial endophytes (TRO4 and CLO5) treated seeds (100%) followed by the chemical (Carbendazim at 2 g kg^−1^ of seeds) treated seeds (93.3%). While the germination percentage was lower in the PLO3-treated seeds (10). The dry plant biomass (g plant^−1^) was higher (1.46) in the PLO3-treated seeds and lower in the uninoculated control (0.74). The plant height (cm) was higher in the TRO4-treated seeds (57.4) and lower in the uninoculated control (40.7). The number of branches per plant varied between 2.5 to 3.0 among the treatments. The MDR and PDI were not recorded from 0 up to 30 DAS as there were no symptoms occurring during this period. At 45 DAS, the PDI was recorded higher in the positive control (13.75) and lower in the PLO3-treated seeds (0.5; Figures 2B, 3A). The PDI of the TRO4 and CLO5 treatments were significantly (*p* ≤ 0.05) lower than the positive control.

#### *Sclerotium rolfsii* pre-challenged soil

In general, the germination percentage was lower in *S. rolfsii* pre-challenged soil. The maximum germination percentage was observed in TRO4-treated seeds (80) and the minimum germination percentage was observed in PLO3-treated seeds (40). The dry plant biomass (g plant^−1^) was higher in the TRO4 treatment (1.32) and lower in the positive control (only pathogen; 0.61). The plant height (cm) was higher in the TRO4-treated seeds (53.8) and lower in the uninoculated control (40.7). The number of branches per plant was higher in TRO4 and CLO5 treated seeds (3.0) and lower in PLO3-treated seeds (2.0). The collar rot disease incidence occurred during the initial stages of plant growth. At 15 DAS, the PDI was higher in the PLO3-treated seeds (37.5) and lower in the TRO4-treated seeds (5). There was no occurrence of disease recorded in the uninoculated control. At 30 DAS, PDI was higher in the PLO3-treated seeds (82.5) and lower in the Carbendazim-treated seeds (10.5). As recorded at 15 DAS, there was no occurrence of disease in the uninoculated control. At 45 DAS, PDI was higher in the PLO3-treated seeds (82.5) and lower in the TRO4-treated seeds (23; *p* ≤ 0.05; Figures 2B, 3B). The PDI recorded in the positive control was 58.25.

#### *F. oxysporum* f.sp. *ciceri* pre-challenged soil

The germination percentage was recorded higher in Carbendazim treated seeds (100) followed by TRO4 treated seeds (93.3) and lower in CLO5 and PLO3 treated seeds (60). The dry plant biomass (g/plant) was recorded higher in the endophytes (TRO4, CLO5, and PLO3) treated seeds compared to the uninoculated control and positive control. The plant height (cm) was recorded higher in the CLO5-treated seeds (51.0) and lower in the uninoculated control (40.7). The number of branches per plant was higher in the CLO5-treated seeds (3.5) and lower in Carbendazim-treated seeds (2.7). At 15 DAS, the PDI was higher in the PLO3-treated seeds (33.3) as compared to the positive control (1.75). No symptoms occurred in the TRO4, CLO5, and uninoculated treatments. Similarly, at 30 DAS, no symptoms occurred in the TRO4, CLO5, and uninoculated treatments whereas a higher PDI was recorded in the PLO3-treated seeds (49.25) as compared to the chemical control (0.5). At 45 DAS, PDI was higher in the PLO3-treated seeds (49.25) and lower in the TRO4-treated seeds (7.5; *p* ≤ 0.05; Figures 2B, 3C). The PDI recorded in the positive control (only pathogen) was 20.75.

### Effect of endophytic *Bacillus subtilis* strains on root growth of chickpea

The effect of inoculation of endophytic *B. subtilis* strains on root growth parameters of chickpea plants pre-challenged with soil-borne fungal pathogens was evaluated. The different root growth parameters estimated were root length (mm), surface area (cm^2^), root diameter (mm), and root volume (cm^3^). The results are given in Table 5.

**Table 5:** Effect of bacterial endophytes inoculation on root parameters of chickpea challenged with soil-borne fungal pathogens.

Treatments	Root length (mm)	Surface area (cm^2^)	Average root dia (mm)	Root volume (cm^3^)
*R. solani* pre-challenged				
UC	156.3^e^	17.9^c^	0.58^a^	0.55^c^
A1	317.0^d^	21.2^c^	0.45^c^	0.63^c^
A2	1056.7^a^	39.4^a^	0.54^a^	1.47^a^
A3	750.0^b^	31.2^b^	0.57^a^	1.27^ab^
A4	403.7^c^	29.8^b^	0.51^b^	1.07^b^
A5	736.7^b^	27.0^b^	0.56^ab^	1.0^b^
CD (0.05%)	66.77	5.37	0.05	0.275
CV	6.58	10.85	5.48	15.49
*S. rolfsii* pre-challenged				
UC	156.3^f^	17.9^c^	0.58^ab^	0.55^d^
B1	310.0^e^	18.7^c^	0.41^d^	0.39^d^
B2	860.0^a^	38.1^a^	0.61^a^	2.03^a^
B3	500.0^b^	26.3^b^	0.53^bc^	1.0^c^
B4	432.0^d^	19.2^c^	0.53^bc^	0.9^c^
B5	463.0^c^	28.3^b^	0.51^c^	1.7^b^
CD (0.05%)	24.13	2.15	0.05	0.24
CV	2.99	4.89	5.5	12.48
*F. oxysporum* f.sp. *ciceri* pre-challenged				
UC	156.3^e^	17.9^e^	0.58^ab^	0.55^d^
C1	278.7^d^	18.5^e^	0.49^c^	0.9^c^
C2	1010.0^a^	37.1^a^	0.57^ab^	2.3^a^
C3	690.0^b^	34.3^b^	0.59^a^	1.5^b^
C4	465.7^c^	28.5^c^	0.52^bc^	1.3^b^
C5	420.6^c^	23.6^d^	0.58^ab^	0.9^c^
CD (0.05%)	57.69	2.38	0.06	0.33
CV	6.44	5.04	6.29	14.82

Treatment details: UC – Uninoculated (negative control). A1, B1, C1 – Positive control of *R. solani, S. rolfsii* and *F. oxysporum* f.sp. *ciceri*, respectively. A2, B2, C2 – Respective pathogen + *B. subtilis* strain TRO4 inoculated. A3, B3, C3 – Respective pathogen + *B. subtilis* strain CLO5 inoculated. A4, B4, C4 – Respective pathogen + *B. subtilis* strain PLO3 inoculated. A5, B5, C5 – Chemical control (Respective pathogen + Carbendazim @ 2 g per kg of seed). The treatment with the same superscript within a column does not differ significantly at *p* = 0.05.

#### *Rhizoctonia solani* pre-challenged soil

In soil pre-challenged with *R. solani*, the root length (mm) was significantly higher (1056.7) in the TRO4-inoculated seeds followed by the CLO5-treated seeds (750) and lower in the uninoculated negative control (156.3). The root surface area (cm^2^) was higher in the TRO4-treated seeds (39.4) followed by the CLO5-treated seeds (31.2) and lower in the uninoculated negative control (17.9). However, the root diameter (mm) was found higher in the uninoculated negative control (0.58) and lower in the positive control (0.45). The root volume (cm^3^) was recorded higher in the TRO4-treated seeds (1.47) followed by the CLO5-treated seeds (1.27) and lower in the uninoculated control (0.55).

#### *Sclerotium rolfsii* pre-challenged soil

In soil pre-challenged with *S. rolfsii*, the root length (mm) was recorded higher in the TRO4-treated seeds (860) followed by the CLO5-treated seeds (500) and lower in the uninoculated control (156.3). The root surface area (cm^2^) was found higher in the CLO5-treated seeds (38.1) followed by the carbendazim-treated seeds (28.3) and lower in the uninoculated control (17.9). The root diameter (mm) was found higher in the TRO4-treated seeds (0.61) followed by the uninoculated control (0.58) and lower in the positive control (0.41). The root volume (cm^3^) was recorded higher in the TRO4-treated seeds (2.03) followed by the carbendazim-treated seeds (1.7) and lower in the positive control (0.39).

#### *Fusarium oxysporum* f.sp. *ciceri* pre-challenged soil

In soil pre-challenged with *F. oxysporum* f.sp. *ciceri*, the root length (mm) was higher in the TRO4-treated seeds (1010) followed by the CLO5-treated seeds (690) and lower in the uninoculated control (156.3). The root surface area (cm^2^) was higher in the TRO4-treated seeds (37.1) followed by the CLO5-treated seeds (34.3) and lower in the uninoculated control (17.9). The root diameter (mm) was higher in the CLO5-treated seeds (0.59) and the uninoculated control (0.58), whereas, root diameter was recorded lower in the positive control (0.49). The root volume (cm^3^) was recorded higher in the TRO4-treated seeds (2.3) followed by the CLO5-treated seeds (1.5) and lower in the uninoculated control (0.55).

### Confocal scanning laser microscopic analysis of endophytic colonization

**Figure 4:**
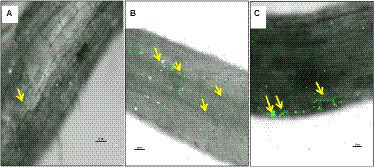
Confocal Scanning Laser Microscopic images indicating colonization of bacterial endophytes in chickpea roots using LIVE/DEAD™BacLight™ Bacterial viability staining, yellow arrows indicating endophytic colonization in the roots. **(A)** Uninoculated control root, **(B)** *B. subtilis* TRO4 inoculated root, **(C)** *B. subtilis* CLO5 inoculated root.

The colonization studies using confocal scanning laser microscopy indicated that root tissues of chickpea plants inoculated with bacterial endophytes (TRO4 and CLO5) produced more signals as compared to the uninoculated control (Figure 4). Confocal microphotograph clearly indicated that the endophyte TRO4 primarily produced micro-aggregates, while CLO5 produced macro-aggregates on the root surface (Figures 4B,C, respectively).

### Effect of endophytic *Bacillus subtilis* strains on induction of ISR in chickpea

The effect of inoculation with endophytic *B. subtilis* strains on the induction of ISR in chickpea plants pre-challenged with soil-borne fungal pathogens was evaluated. The different ISR parameters estimated were the accumulation of superoxide radicals, plant defense enzymes, and the up-regulation of defense-related genes.

#### Accumulation of superoxide radicals

**Figure 5:**
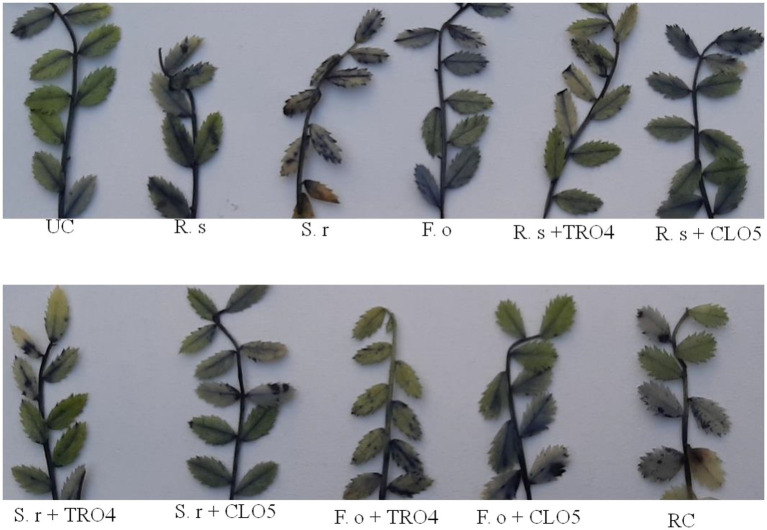
Effect of bacterial endophytes on the accumulation of superoxide radicals in chickpea leaves. UC, Un-inoculated control in which neither pathogen nor bacterial strain inoculated; R.s, *Rhizoctonia solani*; S.r, *Sclerotium rolfsii*; F. o, *Fusarium oxysporum* f. sp. *ciceri*; RC, Resistant cultivar.

**Figure 6:**
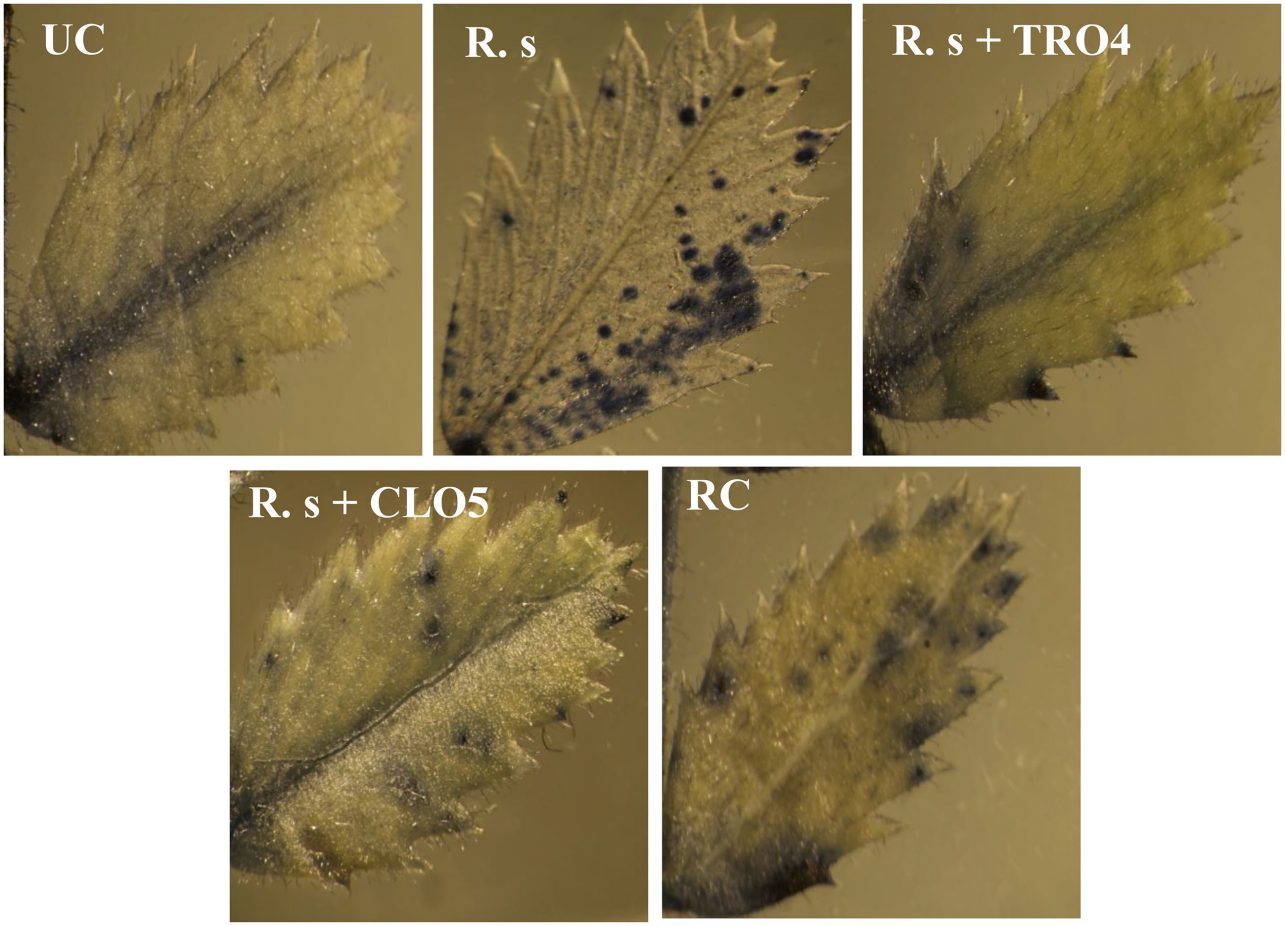
Visualization of accumulation of superoxide radicals in chickpea leaves under a stereomicroscope. UC, Un-inoculated control in which neither pathogen nor bacterial strain inoculated; R.s, *Rhizoctonia solani*; S.r, *Sclerotium rolfsii*; F. o, *Fusarium oxysporum* f. sp. *ciceri*; RC, Resistant cultivar.

Superoxide (O^2−^) accumulation in chickpea leaves was found to vary among the treatments (Figure 5). The visualization of superoxide accumulation in *R. solani* pre-challenged treatments under stereomicroscope is given in Figure 6. NBT staining revealed that the positive control plants (only pathogen challenged) had the highest accumulation of superoxide. The negative control plants had the least accumulation since there was no pathogen inoculation and thus the generation of oxidative stress was also low. Reduced superoxide accumulation was observed in chickpea plants treated with endophytes, TRO4 followed by CLO5.

#### Plant defense enzymes

**Figure 7:**
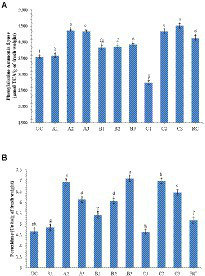
Effect of bacterial endophytes inoculation on plant defense enzymes of chickpea challenged with soil-borne fungal pathogens. **(A)** Phenylalanine Ammonia Lyase; **(B)** Peroxidase. Treatment details: UC-Uninoculated (negative control which is not inoculated with a pathogen or bacterial strain). A1, B1, C1 – Positive control of *R. solani*, *S. rolfsii,* and *F. oxysporum* f. sp. *ciceri*, respectively in which only pathogen inoculated. A2, B2, C2 – Respective pathogen + *B. subtilis* strain TRO4 inoculated. A3, B3, C3 – Respective pathogen + *B. subtilis* strain CLO5 inoculated. RC, Resistant cultivar. Data are mean (*n* = 5) and vertical bars represent standard deviation.

The analysis of defense enzymes Peroxidase (PO) and Phenylalanine Ammonia-Lyase (PAL) in chickpea leaves showed an increased level of enzyme activity in the endophyte-inoculated plants pre-challenged with soil-borne fungal pathogens than the positive control (only pathogen challenged; Figure 7). The highest activity of PAL (4513; μmol TCA g^−1^ of fresh weight) was recorded in the CLO5-treated chickpea plants pre-challenged with *F. oxysporum* f.sp. *ciceri* while the lowest activity (3537) was observed in the uninoculated plants (negative control). Similarly, the highest PO activity (7.09; units g^−1^ of fresh weight) was observed in the CLO5-treated chickpea plants pre-challenged with *S. rolfsii* while the lowest activity (4.64) was observed in the *F. oxysporum* f.sp. *ciceri* pre-challenged plants.

#### Up-regulation of defense-related genes

**Figure 8:**
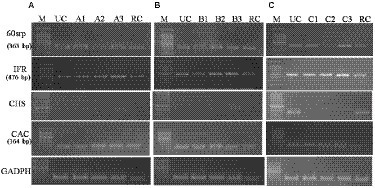
Effect of bacterial endophytes inoculation on expression of PR genes (**A**, *R. solani* pre-challenged; **B**, *S. rolfsii* pre-challenged; **C**, *F. oxysporum* f. sp. *ciceri* pre-challenged) Treatment details: UC-Uninoculated (negative control in which neither pathogen nor bacterial strain inoculated). A1, B1, C1 – Positive control of *R. solani*, *S. rolfsii,* and *F. oxysporum* f. sp. *ciceri*, respectively in which only pathogen inoculated. A2, B2, C2 – Respective pathogen + *B. subtilis* strain TRO4 inoculated. A3, B3, C3 – Respective pathogen + *B. subtilis* strain CLO5 inoculated. RC, Resistant cultivar.

Semi-quantitative RT-PCR analysis of defense-related genes was performed using gene-specific oligonucleotides in endophytes-inoculated and pathogen-challenged chickpea plants (cv. JG-62; Figure 8). A resistant cultivar (var. avrodhi) was used as a standard check. The expression of PR genes, *60srp* (gene for 60s ribosomal protein), and *IFR* (Isoflavone reductase) were studied with the normalization of *CAC* (Clathrin adaptor complexes medium subunit family protein). The stable expression of the housekeeping gene, *CAC,* was noticed in all the treatments. The results showed the expression of the *60srp* gene was highest in endophyte-inoculated and pathogen-inoculated plants than in pathogen-alone and resistant cultivars. The expression of the *60srp* gene was higher in the CLO5-inoculated and plants challenged with *F. oxysporum* f.sp. *ciceri*. In general, the expression level of the *IFR* gene was higher in endophyte-inoculated and pathogen-challenged plants. The increase in expression of the IFR gene was noticed in the CLO5-inoculated plants and plants challenged with *F. oxysporum* f.sp. *ciceri*.

## Discussion

The irrational use of chemical fungicides in agriculture causes serious health problems to humans and animals along with irreparable deleterious effects on the environment. Researchers worldwide have undertaken research on environmentally friendly agents as an alternative to toxic chemical fungicides. Biocontrol is one of the strategies for the eco-friendly management of crop diseases. It uses naturally occurring bio-agents such as viruses, bacteria, fungi, algae, insects, etc., to control crop diseases and pests, and endophytes are one of them (Bacon and White, 2000). Bacterial endophytes are a group of bacteria that reside inside the root, stem, leaves, and other parts of a plant. They are potential bio-agents as they provide localized protection to the host plant against the invasion of pathogenic fungi and bacteria (Sahu et al., 2020; Singh et al., 2020a). They produce antimicrobial compounds, provide systemic resistance to the host plant and improve plant growth by solubilizing the mineral nutrients (phosphorus, potassium, zinc etc.), fixing atmospheric nitrogen, producing siderophore, IAA, etc. (Ryan et al., 2007). Previously, several attempts have been made to isolate bacterial endophytes from different crops such as Rice, Maize, Peanut, Banana, Soybean, Medicinal crops, etc. (Souza et al., 2014; Gond et al., 2015; Zhao et al., 2015). In the present study, we have identified three potential bacterial endophytes (TRO4, CLO5, and PLO3) out of 255, based on the screening of antagonistic activity against all the three fungal pathogens (*R. solani*, *S. rolfsii*, and *F. oxysporum* f.sp. *ciceri*). The molecular identification of these three endophytes revealed that they are different strains of *Bacillus subtilis*. The results are in agreement with the previous reports where most of the potential bacterial endophytes as a biocontrol agent were identified as *Bacillus* and *Pseudomonas* irrespective of the crops used for isolation (Sun et al., 2013; Chen et al., 2014; Zhao et al., 2015; Kumar et al., 2016; Vinodkumar et al., 2017). In a similar study, the inhibition percentage of soil-borne fungal pathogens by potent bacterial endophytes was 50 to 70% (Sahu et al., 2019). The endophytic *Bacillus subtilis* has also been reported in other studies for the control of *Fusarium* and other plant pathogens through the production of diffusible and volatile inhibitory compounds (Hazarika et al., 2019). The usefulness of endophytic *Bacillus subtilis* was also proven in the biocontrol of take-all disease in wheat in which the bacterium was found to have endophytic colonization and disintegrate the cytoplasm of the fungal pathogen (Chung et al., 2008; Liu et al., 2009).

The biocontrol efficacy of selected bacterial endophytes against the wilt complex disease in chickpea plants was evaluated under pot culture conditions in soil pre-challenged with fungal pathogens, i.e., *R. solani*, *S. rolfsii*, and *F. oxysporum* f.sp. *ciceri*. The results revealed that the incidence of collar rot caused by *S. rolfsii* occurred in the initial stage whereas the incidences of root rot caused by *R. solani* and fungal wilt caused by *F. oxysporum* f.sp. *ciceri* occurred at later stages. A similar observation was made in the evaluation of *Trichoderma* in suppressing wilt complex disease in chickpea plants (Rudresh et al., 2005). The results of the pot experiment showed that the chickpea plants inoculated with bacterial endophytes (TRO4 and CLO5) had a lower PDI as compared to pathogen alone (positive control; Figures 2, 3). Similarly, the vigor, plant growth characteristics, and germination percentage in chickpea plants were higher in TRO4 and CLO5 treated seeds. Similar results on the effectiveness of bacterial endophytes in increasing the germination percentage, vigor, and plant growth characteristics were reported in tomato plants challenged with *S. rolfsii* (Sahu et al., 2019) and rice plants challenged with *R. solani* (Sahu et al., 2020). In a similar experiment, the disease control percentage (60–65) of wilt complex in chickpea plants was recorded in seeds treated with *T. viride* at 8 g kg^−1^ or soil applied with *T. harzianum* (Kaur and Mukhopadhyay, 1992; Rajput et al., 2010). The different root growth parameters, root length (mm), surface area (cm^2^), root diameter (mm), and root volume (cm^3^) were significantly (*p* ≤ 0.05) improved in TRO4 and CLO5 inoculated chickpea plants. The improved root growth parameters provide the host plant resistance against the invasion of soil-borne fungal pathogens. These results are in agreement with Singh et al. (2017) where inoculation with zinc solubilizing bacteria, *B. subtilis* DS-178 and *Arthrobacter* sp. DS – 179, improved the root growth parameters in wheat.

Bacterial endophytes able to successfully colonize the plant tissue could impart protection from biotic and abiotic stresses to the host plants. The analysis of the root tissue of chickpea plants using confocal microscopy revealed that the endophytic *B. subtilis* strains TRO4 and CLO5 inoculated plants produced more signals than the control. The uninoculated control plants also showed weak signals which might be due to their native endophytes (Figure 4). Following the dye system used for staining bacteria (Boulos et al., 1999), the intensity of the signals is proportional to the bacterial cells colonizing the root tissue which indicates successful colonization of the bacterial endophytes in the plant roots. The results are in agreement with other researchers (Sahu et al., 2020). The induction of systemic resistance in chickpea plants pre-challenged with soil-borne fungal pathogens by seed priming with bacterial endophytes was evaluated. Bacterial endophytes stimulate the expression of PR genes and phenol content and increase the activity of PR proteins such as PO, PPO, PAL, chitinases, lipoxygenases, and glucanases in the host plant as a defense mechanism to suppress the pathogenic effect (Zaim et al., 2016; Bekkar et al., 2018; Kumari and Khanna, 2019). In the present study, we examined the accumulation of superoxide radicals, defense-related enzymes (PO and PAL), and the up-regulation of defense-related genes (*60srp* and *IFR*) to evaluate bacterial endophytes for the induction of systemic resistance in chickpea plants pre-challenged with soil-borne fungal pathogens.

Reactive oxygen species (ROS), i.e., ^1^O_2_, O^2−^, H_2_O_2_, and OH^−^, are generated as signaling molecules in biotic and abiotic stressed plants and an excess amount of these ROS is deleterious to plant growth (Czarnocka and Karpiński, 2018). The antioxidant machinery of plants keeps the levels of superoxide and other ROS under control. There are reports of endophytes supplementing enzymatic and non-enzymatic antioxidant production in the host plants (Sahu et al., 2019, 2020). The reduced accumulation in TRO4 and CLO5 inoculated treatments indicate protective effects against superoxide accumulation at deleterious levels under biotic stress conditions (Figures 5, 6). Bacterial endophytes are able to induce the expression of defense-related enzymes such as PAL, PO, polyphenol oxidases (PPO), and phenols in the host plant to enable defense against invading pathogens. The activity of defense enzymes PAL and PO were higher in the CLO5-treated plants than in the positive control (pathogen alone; Figure 7). In a similar study, chickpea plants pre-challenged with *F. oxysporum* f.sp. *ciceri* and inoculated with the isolates of antagonistic rhizobacteria (Ps 45 and Ba1a) along with native *Mesorhizobium* exhibited the highest activity of PAL, PO, and PPO compared to fungicide treatment and the positive control (pathogen alone; Kumari and Khanna, 2019; Malviya et al., 2020). PAL and PO are the key enzymes of phenylpropanoid pathways. PAL is a key/primary enzyme of the phenylpropanoid pathway which catalyzes the formation of intermediate metabolites such as cinnamic acid, p-coumaric acid, caffeic acid, ferulic acids, and gallic acid which ultimately leads to cell wall lignification and thickening (Lopez and Hernández, 2014). Phenylpropanoids are one of the important cascades activated during host-pathogen interaction. They regulate a wide range of physiological processes inside the plant cells and modulate the expression of key genes in the plants, inducing ISR and SAR against biotic stresses across the plant species (Singh A. et al., 2013; Singh U. B. et al., 2013; Lopez and Hernández, 2014). Our results are in accordance with the findings of Harman et al. (2004) and Singh et al. (2013), who observed that the inoculation of plants with *T. harzianum*, *B. amyloliquefaciens,* and other PGPRs induced cascades relating to plant defense and the up-regulation of enzymes over a period of time. In general, PAL and PO elicit several downstream processes leading to defense responses characterized by the inhibition of growth of invading fungi through phytoalexin formation, callose deposition, cell wall lignifications, synthesis of antimicrobial secondary metabolites, and pathogenesis-related (PR) proteins (Harman et al., 2004; Lopez and Hernández, 2014). Cell wall lignification renders the plant more resistant to pathogen attack (Tiwari et al., 2011; Jain et al., 2012; Lopez and Hernández, 2014). The bacterial inoculants induce the expression of PR genes in the host plant to overcome the deleterious effects of biotic stress caused by the invading pathogens. In our study, we observed the up-regulation of expression of PR genes in endophytes inoculated plants. According to Reddy et al. (2016), the genes *UCP* and *G6PD* were stably expressed while *TIP41* and *CAC* were found to be highly stable in the chickpea genotypes. In this study, *CAC* was chosen as the housekeeping gene and used for normalization. The expression of the PR genes (*60srp* and *IFR*) was noticed to be highly up-regulated in chickpea plants pre-challenged with *F. oxysporum* f.sp. *ciceri* and bio-primed with CLO5 compared to the resistant check and positive control (pathogen alone). These observations clearly indicated the protection afforded to host plants by bacterial endophytes under pathogenic stressed conditions (Figure 8). Similar observations were made by Gurjar et al. (2012). The present study revealed the up-regulation of defense-related genes and the over-expression of PR proteins in chickpea plants bio-primed with bacterial endophytes and their role in combating pathogenesis in the host plants under biotic stress conditions. Overall, the results suggested that the application of TRO4 and CLO5 not only helps control wilt complex disease but also increased plant growth, as well as enhances the systemic resistance of chickpea plants against plant pathogens causing wilt complex.

## Conclusion

The bacterial endophytes isolated in the study were found to have potential biocontrol activity. *In-vitro* experiments indicated three promising isolates have antagonistic activity against all three soil-borne fungal pathogens (*R. solani, S. rolfsii,* and *F. oxysporum* f.sp. *ciceri*). The *in-planta* assay revealed that TRO4 and CLO5 treated seeds had improved plant growth and reduced disease incidence percentage of wilt complex disease in chickpea plants. The current study provides insight into the possibilities of using potential endophytes for managing wilt complex disease in chickpea plants. Such an approach would be an eco-friendly means to manage wilt disease and would also contribute to the maintenance of plant and soil health.

## Data availability statement

The datasets presented in this study can be found in online repositories. The names of the repository/repositories and accession number(s) can be found in the article/Supplementary material.

## Author contributions

VM: conceptualization and writing-original draft preparation. VM, PS, and US: methodology. HS: validation. VM, HC, and SB: formal analysis. RG and SS: investigation. HS: resources. PS and SP: writing-review and editing. All authors contributed to the article and approved the submitted version.

## Funding

The funding received from ICAR-NBAIM, Mau, Uttar Pradesh, India to carry out the present study is acknowledged.

## Conflict of interest

The authors declare that the research was conducted in the absence of any commercial or financial relationships that could be construed as a potential conflict of interest.

## Publisher’s note

All claims expressed in this article are solely those of the authors and do not necessarily represent those of their affiliated organizations, or those of the publisher, the editors and the reviewers. Any product that may be evaluated in this article, or claim that may be made by its manufacturer, is not guaranteed or endorsed by the publisher.
