# Supplementary material for: Endophytic Bacillus subtilis antagonize soil-borne fungal pathogens and suppress wilt complex disease in chickpea plants (Cicer arietinum L.)
Source: Front Microbiol. 2022 Nov 2;13:994847. doi: 10.3389/fmicb.2022.994847 (PMC9667066; doi:10.3389/fmicb.2022.994847)
Supplement: Supplementary file 1 [file Data_Sheet_1.docx]

**Supplementary Tables**

**Endophytic *Bacillus subtilis* antagonize soil-borne fungal pathogens and suppress wilt complex disease in chickpea plants (*Cicer arietinum* L.)**

V. Mageshwaran^1*^, Rishabh Gupta^1^, Shailendra Singh^2^, Pramod K. Sahu^2^, Udai B. Singh^2*^, Hillol Chakdar^1^, Samadhan Y Bagul^3^, Surinder Paul^1^ and Harsh V. Singh ^2^

^1^ ICAR- National Bureau of Agriculturally Important Microorganisms, Kushmaur, Maunath Bhanjan 275 103, Uttar Pradesh, India

^2^ Plant-Microbe Interaction and Rhizosphere Biology Lab, ICAR-National Bureau of Agriculturally Important Microorganisms, Kushmaur, Maunath Bhanjan 275 103, Uttar Pradesh, India

^3^ ICAR-Directorate of Medicinal and Aromatic Plants Research, Boriavi, Anand 387 310, Gujarat, India

**Supplementary Table 1.** Effect of endophytes inoculation on disease incidence in chickpea

| Treatments | 15 DAS |  | 30 DAS |  | 45 DAS |  |
| --- | --- | --- | --- | --- | --- | --- |
|  | MDR | PDI | MDR | PDI | MDR | PDI |
| UC | 0 | 0 | 0 | 0 | 0.22 | 5.5 |
| *R. solani* pre-challenged | | | | | | |
| A1 | 0 | 0 | 0 | 0 | 0.55 | 13.75 |
| A2 | 0 | 0 | 0 | 0 | 0.3 | 7.5 |
| A3 | 0 | 0 | 0 | 0 | 0.3 | 7.5 |
| A4 | 0 | 0 | 0 | 0 | 0.2 | 0.5 |
| A5 | 0 | 0 | 0 | 0 | 0.4 | 10 |
| *S. rolfsii* pre-challenged | | | | | | |
| B1 | 0.9 | 22.5 | 1.66 | 41.65 | 2.33 | 58.25 |
| B2 | 0.2 | 5 | 0.75 | 18.75 | 0.92 | 23 |
| B3 | 0.83 | 20.8 | 2.13 | 53.25 | 2.67 | 66.75 |
| B4 | 1.5 | 37.5 | 3.3 | 82.5 | 3.3 | 82.5 |
| B5 | 0.25 | 6.25 | 0.42 | 10.5 | 1.55 | 38.75 |
| *F. oxysporum* f. sp. *ciceri* pre-challenged | | | | | | |
| C1 | 0.07 | 1.75 | 0.2 | 0.5 | 0.83 | 20.75 |
| C2 | 0 | 0 | 0 | 0 | 0.3 | 7.5 |
| C3 | 0 | 0 | 0 | 0 | 0.38 | 9.5 |
| C4 | 1.3 | 33.3 | 1.97 | 49.25 | 1.97 | 49.25 |
| C5 | 0.06 | 1.66 | 0 | 0 | 0.4 | 10 |

**Note:**DAS- Days After Sowing; MDR- Mean Disease Rate; PDI – Percent Disease Incidence. Treatment details: UC- Uninoculated (negative control). A1, B1, C1 – Positive control of *R. solani*, *S. rolfsii,* and *F. oxysporum* f. sp. *ciceri* respectively. A2, B2, C2- Respective pathogen + *B. subtilis* strain TRO4 inoculated. A3, B3, C3- Respective pathogen + B*. subtilis* strain CLO5 inoculated. A4, B4, C4- Respective pathogen + *B. subtilis* strain PLO3 inoculated. A5, B5, C5- Chemical control (Respective pathogen + Carbendazim at 2 g per kg of seed).

**Supplementary Table 2.** Effect of endophytes inoculation on plant growth in chickpea

| Treatments | Germination percentage | Dry plant biomass (g/plant) | Plant height (cm) | No. of branches per plant |
| --- | --- | --- | --- | --- |
| UC | 66 | 0.74 | 40.7 | 2.8 |
| *R. solani* pre-challenged | | | | |
| A1 | 80 | 0.88 | 49.7 | 2.6 |
| A2 | 100 | 0.92 | 57.4 | 3.0 |
| A3 | 100 | 1.06 | 50.4 | 2.8 |
| A4 | 10 | 1.46 | 51.0 | 3.0 |
| A5 | 93.3 | 0.91 | 52.0 | 2.5 |
| *S. rolfsii* pre-challenged | | | | |
| B1 | 50 | 0.61 | 45.5 | 2.2 |
| B2 | 80 | 1.32 | 53.8 | 3.0 |
| B3 | 60 | 1.1 | 45.5 | 3.0 |
| B4 | 40 | 0.84 | 45.0 | 2.0 |
| B5 | 53.3 | 1.16 | 48.9 | 2.7 |
| *F. oxysporum* f. sp. *ciceri* pre-challenged | | | | |
| C1 | 80 | 0.86 | 48.9 | 3.2 |
| C2 | 93.3 | 0.97 | 46.8 | 3.3 |
| C3 | 60 | 1.1 | 51.0 | 3.5 |
| C4 | 60 | 1.04 | 48.1 | 3.0 |
| C5 | 100 | 0.89 | 46.5 | 2.7 |

**Note:**Treatment details: UC- Uninoculated (negative control). A1, B1, C1 – Positive control of *R. solani*, *S. rolfsii,* and *F. oxysporum* f. sp. *ciceri* respectively. A2, B2, C2- Respective pathogen + *B. subtilis* strain TRO4 inoculated. A3, B3, C3- Respective pathogen + B*. subtilis* strain CLO5 inoculated. A4, B4, C4- Respective pathogen + *B. subtilis* strain PLO3 inoculated. A5, B5, C5- Chemical control (Respective pathogen + Carbendazim at 2 g per kg of seed).

**Supplementary Table 3.** Primer sequence of chickpea defense related proteins

| **S.No.** | **Gene** | **Forward primer sequence** | **Reverse primer sequence** | **Product length (bp)** |
| --- | --- | --- | --- | --- |
| 1. | CAC (Clathrin adaptor complexes medium subunit family protein)-Reference gene | CATGGACTAGACCACCAATTCA | AACAGTGTTGTACCCGCTCTTT | 164 |
| 2. | 60srp (gene for 60s ribosomal protein) | GATCAGGGGAATCGGAAAAT | GACTGATTCACACGCCTCAA | 363 |
| 3. | IFR (Isoflavone reductase) | GCTGCTGCTAACCCTGAAAG | GTTCCCACATCAGCCTCAGT | 476 |
